# Gastrointestinal Stromal Tumors of Small Intestine

**DOI:** 10.1055/s-0039-1694704

**Published:** 2019-08-28

**Authors:** Tanweerul Huda, Mahendra Pratap Singh

**Affiliations:** 1 Department of General Surgery, All India Institute of Medical Sciences (AIIMS), Bhopal, Madhya Pradesh, India

**Keywords:** gastrointestinal tumor, gastrointestinal stromal tumors, jejunal tumor, CD117, CD34, PDGFRA, imatinib

## Abstract

Gastrointestinal stromal tumor (GIST) is defined as mesenchymal tumors of the gastrointestinal tract expressing proto-oncogene protein CD117. They are the most common sarcomatous tumors of the gastrointestinal tract. GISTs are presumed to arise from interstitial cells of Cajal or gastrointestinal pacemaker cells which control gut motility. They have unpredictable biological behavior. Prognosis is dependent on tumor size as well as mitotic count. Radical surgical excision is the treatment of choice. They rarely metastasize to lymph nodes. Imatinib therapy is used as an adjuvant therapy. The follow-up of patients postsurgery is not standardized.

Gastrointestinal stromal tumors (GISTs) are defined as mesenchymal tumors of the gastrointestinal (GI) tract expressing proto-oncogene protein CD117. 1 They are the most common sarcomatous tumors of the GI tract. They were originally believed to be a smooth muscle sarcoma and previously named as leiomyoma and leiomyosarcoma. 1 They are now known to originate from Interstitial cells of Cajal (Intestinal pacemaker cells). They are equally observed in males and females. 1 They can manifest at any age, more commonly in patients older than 50 years. Some cases in children and young adults have also been reported. 2 They can appear anywhere in the GI tract, stomach (40–60%), jejunum and ileum (30%), duodenum (5%), colon (15%), and very rarely in the esophagus and appendix. Extra-GI tract GISTs have been reported in the omentum, mesentery, retroperitoneum, gallbladder, and urinary bladder. 3 4 5 More than 95% of GISTs express CD117 (a c-kit proto-oncogene), while 70 to 90% express CD34 (human progenitor cell antigen). Along with the c-kit, platelet-derived growth factor receptor alpha (PDGFRA) mutations (exon 18) are also seen. The c-kit mutations are seen in exon 9 and 11. These tumors may sometimes stain positive for actin (20–30%), S100 (2–4%), and desmin (2–4%). GISTs vary considerably in their presentation and clinical course. Most GISTs are asymptomatic, and diagnosed incidentally when doing an abdominal radiological investigation or during a surgery for another etiology. Symptoms depend on the size and location of the tumor. Symptomatic GISTs usually present with bleeding (hematemesis/melena), vague abdominal pain or discomfort, and weight loss. These tumors may show intramural growth leading to obstruction or have intramural and extramural growth leading them to achieve massive size. Some patients with large GISTs may have externally palpable masses. 6 7 Lymph node metastasis is extremely rare. 1 The most common metastasis is to the peritoneum and liver. 1 Metastasis to the lung and bone in some cases has been reported. 8 Size and mitotic index are the best predictors of metastasis. Mitotic index is classified as low (less than 5 mitoses/50 high-power fields [hpf]) or high (more than 5 mitoses/50 hpf). Patients are evaluated using upper GI endoscopy, endoscopic ultrasound (EUS), and contrast-enhanced computed tomography (CECT) abdomen and pelvis (to assess metastasis). The upper GI endoscopic picture shows a smooth, round, submucosal tumor with central ulceration. Surgery is the primary modality of treatment.

## Materials and Methods

### Case Presentation

**Fig. 1:**
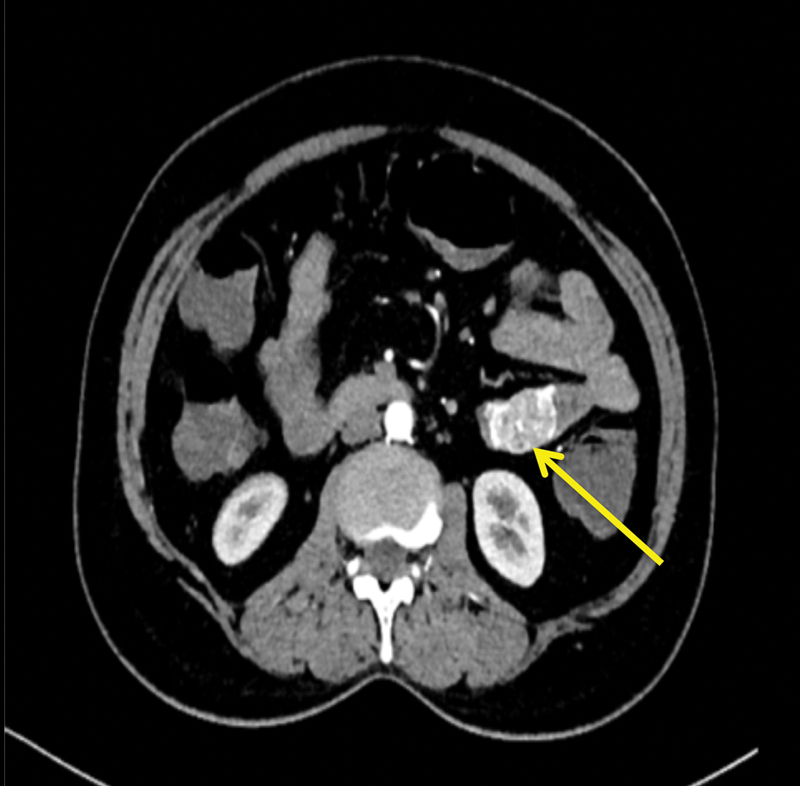
Contrast-enhanced computed tomography (CECT) showing jejunal gastrointestinal stromal tumor (GIST).

**Fig. 2:**
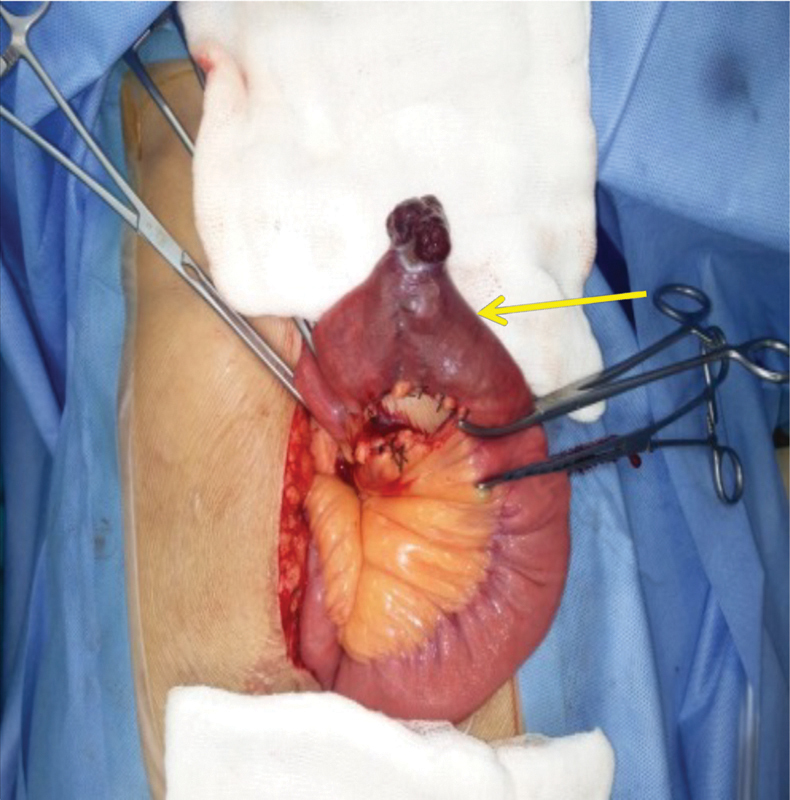
Gastrointestinal stromal tumor (GIST) in the jejunum as exophytic lesion.

**Fig. 3:**
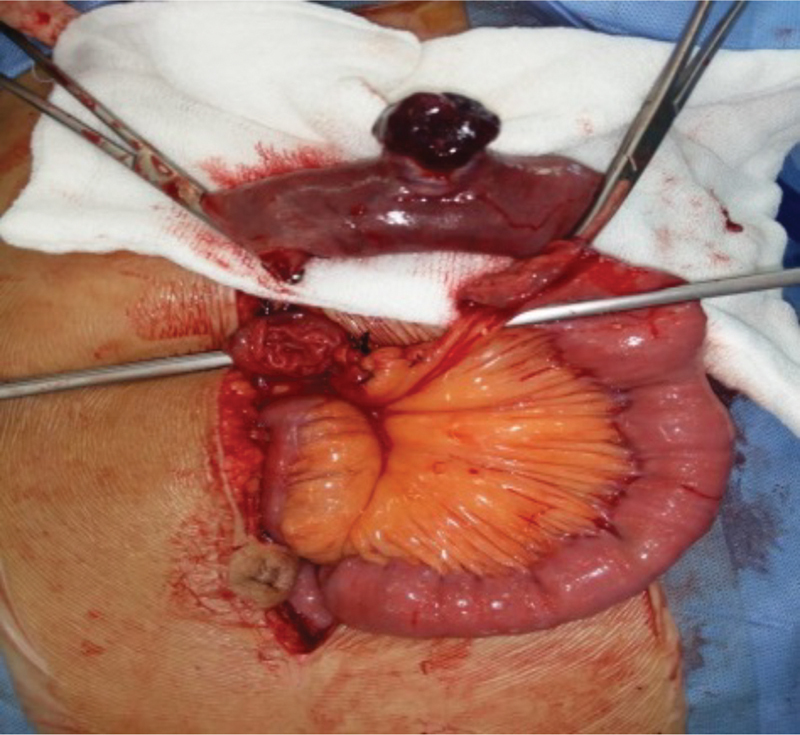
Resected gastrointestinal stromal tumor (GIST) with 5 cm margin.

**Fig. 4:**
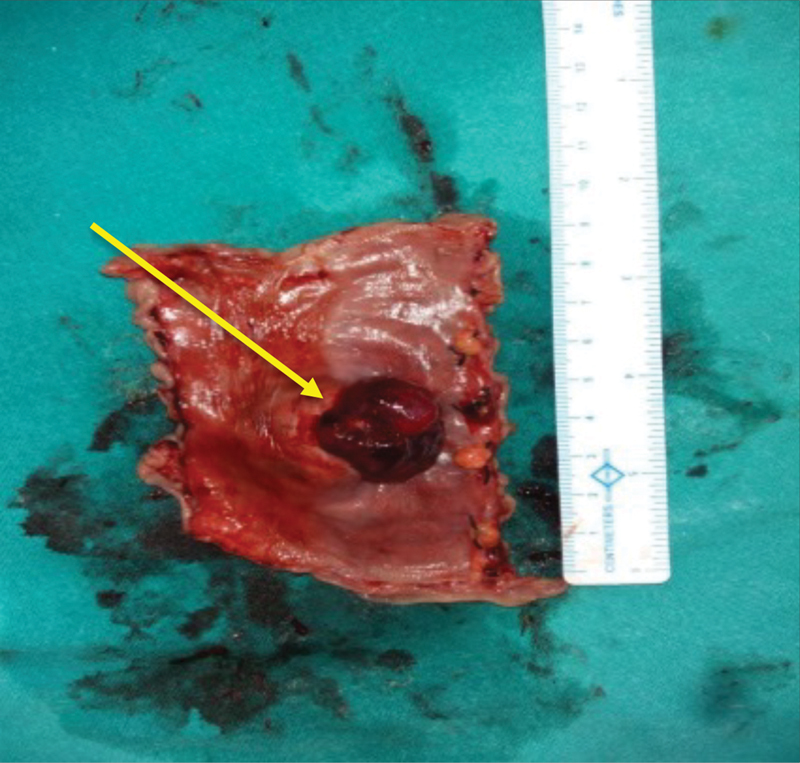
View of jejunum showing serosal breach.

**Fig. 5:**
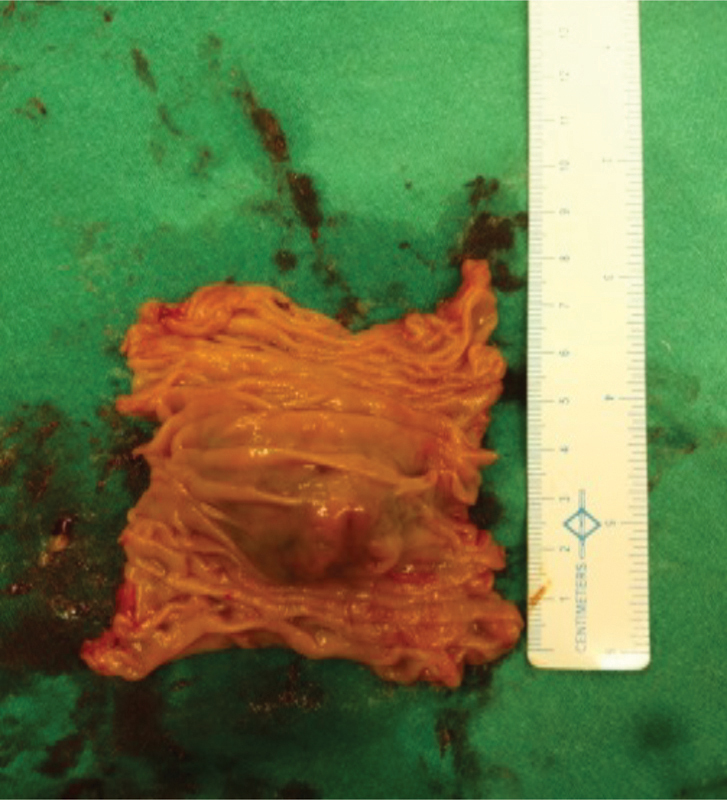
Cut section of jejunum showing intact mucosa.

**Fig. 6:**
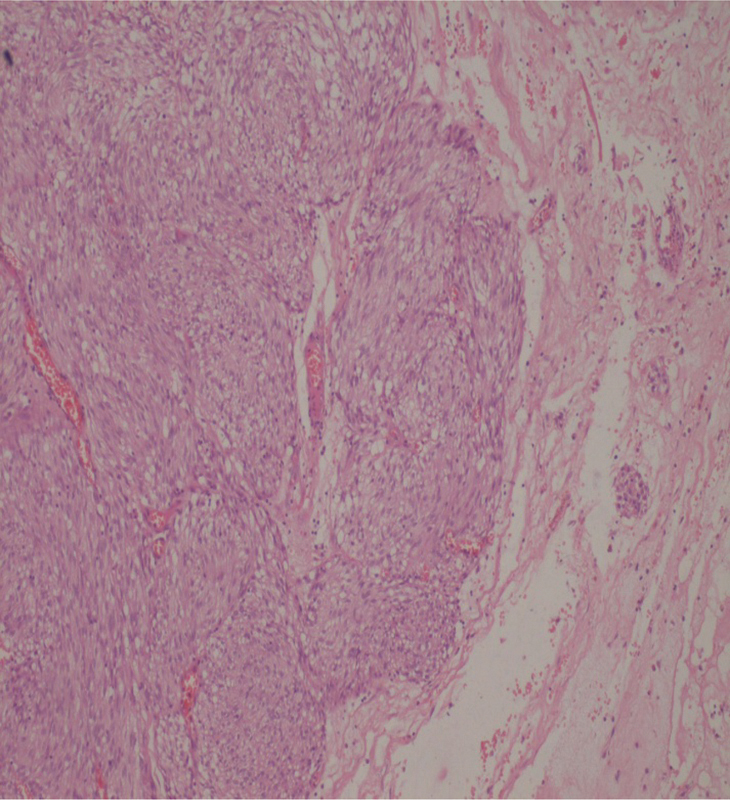
Photomicrograph showing spindle cells arranged in fascicles and interlacing bundles. Cells had oval to elongated vesicular to hyperchromatic nucleus with inconspicuous nucleoli and cytoplasm in moderate amount. Occasional mitosis, thin walled blood vessels. Interspersed in between the lesion cells are foci of hemorrhage and fibrin deposition.

A 55-year-old, hypertensive female patient presented with the chief complain of pain in the abdomen for the past 4 years, which was dull in nature, and not associated with any fever, vomiting, diarrhea, constipation, or any urinary complains. There was an associated history of loss of appetite but no history of weight loss. There was no associated history of hematemesis or melena. Patient was taking treatment on and off but was not relieved. Perabdomen examination revealed a lump in the umbilical region. All routine laboratory investigations were unremarkable. Upper GI endoscopy was unremarkable. CECT abdomen ( Fig. 1 ) revealed a well-defined avidly enhancing mass lesion arising from the wall of proximal jejunum with locoregional extension. The lesion was causing partial luminal narrowing without any evidence of bowel obstruction. Features were suggestive of neuroendocrine tumor/GI tumor in the jejunum. The patient was taken up for exploratory laparotomy and a tumor was found in the proximal jejunum around 40 cm from duodenojejunal junction ( Fig. 2 ), and without any local extension or any evidence of mesenteric lymphadenopathy. The jejunal segment was resected taking a 5-cm margin from both sides ( Fig. 3 ), followed by end-to-end jejunojejunal anastomosis. Postoperative stay was uneventful. The cut surface showed the lesion breaching the serosa ( Fig. 4 ), whereas no breach in the mucosa was seen ( Fig. 5 ). Histopathological examination report revealed a lesion composed of spindle cells arranged in fascicles and interlacing bundles. Cells had oval to elongated vesicular to hyperchromatic nucleus with inconspicuous nucleoli and cytoplasm in moderate amount. Occasional mitosis, thin walled blood vessels. Interspersed in between the lesion cells are foci of hemorrhage and fibrin deposition ( Fig. 6 ). Immunohistochemistry report showed smooth muscle antibody positive, DOG1 positive, CD 117 focally positive, CD 34 negative, and K _i_ -67 of 2%. Features were suggestive of GIST.

## Results

The histopathological examination report revealed a R0 resection. Imatinib therapy was started and continued for 3 years. Patient was followed up for 3 years postoperatively with CECT abdomen every 6 months. There was no evidence of any recurrence in the follow-up. No specific GI symptoms were seen.

## Discussion

GISTs are tumors with unpredictable biological behavior.

Three syndromes are linked to GISTs:

- Carney triad syndrome, comprising of gastric GISTs, paraganglioma, and pulmonary chondromas. 9
- Carney–Stratakis syndrome, comprising of GIST and paraganglioma. 10 11
- Neurofibromatosis type 1 (NF1), comprising multifocal GIST, mostly located in the small intestine. 12

Grossly, the stromal tumors are firm, gray white lesions having a whorled appearance on cut surface. Microscopically well-differentiated smooth muscle cells are seen. Three histological types are seen on the basis of cellular appearance, fusiform (77%), epithelioid (8%), and mixed (15%). Diagnosis is confirmed by biopsy with immunohistochemistry positive for CD117, CD34, or PDGFRA. Some GISTs express positivity for DOG1 also. The mainstay of treatment is complete surgical resection. Tumors greater than 2 cm in diameter should be resected. Tumors less than 2 cm with high-risk features like irregular borders, ulceration, etc. should also be resected, whereas without these features can be observed with repeated upper GI endoscopy and EUS every 6 months. Smaller tumors can be treated by wedge resection, whereas larger tumors may require a gastrectomy/duodenectomy. 1 The aim of surgery should be to have an R0 resection. No lymph node dissection is required as lymph node metastasis is rare. Recurrence rates are around 40% and most patients demonstrate metastasis to liver, while only one-third of the patients have isolated local recurrence. Long-term disease-free survival is around 50%. Gastric GISTs have a more favorable prognosis than other GISTs. The risk factors for malignancy and recurrence are tumors larger than 10 cm and having more than 5 mitoses/50 hpf. Most benign tumors have low mitotic index (less than 5 mitoses/50 hpf). Adjuvant therapy with imatinib (tyrosine kinase receptor inhibitor) is used to prevent recurrence following surgery, in unresectable cases and with metastatic diseases. It is effective in patients with GISTs who have mutations in exon 11 of the kit gene. Patients having mutations in exon 9 of kit gene also may respond to imatinib but with higher doses, whereas patients without mutations in the kit gene do not respond to imatinib. 13 The Scandinavian Sarcoma Group XVIII trial established a postoperative therapy of 3 years with imatinib in high-risk GISTs. 13 Sunitinib (tyrosine kinase inhibitor) is used for treatment of imatinib refractory GIST and in patients unable to tolerate imatinib. 13 Sunitinib targets multiple kinases, including vascular endothelial growth factor receptors, PDGFRA, KIT, and FLt3. Other newer drugs in development are sorafenib, dasatinib, nilotinib, and regorafenib. 13 18-Fluoro-deoxyglucose-positron emission tomography is widely being accepted now for preoperative screening of GISTs as well as detecting early response to imatinib. 13

The optimal follow-up criteria for patients of GISTs need further studies. Very low-risk GISTs may not require routine follow-up. For low-risk tumors, follow-up may be done with abdominal CT scan or magnetic resonance imaging (MRI), every 6 to 12 months for 5 years. High-risk patients require follow-up with an abdominal CT scan or MRI every 3 to 6 months for 3 years during adjuvant therapy, then on cessation of adjuvant therapy every 3 months for 2 years, then every 6 months until 5 years from stopping adjuvant therapy, and annually for an additional 5 years. 14

## Conclusion

*Diagnostics* : 13 In case of size less than 2 cm, in esophagus, stomach, and duodenum, regular EUS should be done. In case of tumors more than 2 cm in size, a biopsy or excision biopsy is to be considered. A mutational analysis should also be done in every GIST case, excluding less than 2 cm size nonrectal GISTs. *Locoregional disease management* : 13 Preferred treatment protocol of a local GIST tumor is complete excision. All attempts should be made to do a R0 resection surgically. R1 resection should only be merited in case of major morbidity of the patient with an R0 resection. Imatinib therapy as an adjuvant to be given for 3 years to patients having higher chances of relapse or in case of rupture during surgery. No role of imatinib in patients having PDGFRA mutation, NF1, and succinate dehydrogenase negative GISTs. Imatinib can be started preoperatively when R1 resection is expected. *Metastatic disease management* : 13 All metastatic and locally advanced GISTs are treated with imatinib as a 400-mg daily dose. In case of KIT 9 mutation, dose is increased to 800 mg daily. Imatinib therapy should be continued lifelong, except in cases of poor patient compliance. Dose of imatinib is modified to 800 mg daily in cases of advancement of the disease while on the same. The second line therapy is with sunitinib, for patients not sensitive/poor compliance with imatinib. The third line therapy is regorafenib, in a dose of 160 mg for 3 weeks every 4 weeks. Reserved only for nonresponders to imatinib and sunitinib. Surgical excision can be considered in nonprogression of disease with imatinib therapy.
